# Emotion and cognition interactions in PTSD: a review of neurocognitive and neuroimaging studies

**DOI:** 10.3389/fnint.2012.00089

**Published:** 2012-10-09

**Authors:** Jasmeet P. Hayes, Michael B. VanElzakker, Lisa M. Shin

**Affiliations:** ^1^National Center for PTSD, VA Boston Healthcare SystemBoston, MA, USA; ^2^Department of Psychiatry, Boston University School of MedicineBoston, MA, USA; ^3^Department of Psychology, Tufts UniversityMedford, MA, USA; ^4^Department of Psychiatry, The Massachusetts General HospitalBoston, MA, USA

**Keywords:** neuropsychology, fMRI, amygdala, threat bias, cognitive control, memory, anxiety, neuroimaging

## Abstract

Posttraumatic stress disorder (PTSD) is a psychiatric syndrome that develops after exposure to terrifying and life-threatening events including warfare, motor-vehicle accidents, and physical and sexual assault. The emotional experience of psychological trauma can have long-term cognitive effects. The hallmark symptoms of PTSD involve alterations to cognitive processes such as memory, attention, planning, and problem solving, underscoring the detrimental impact that negative emotionality has on cognitive functioning. As such, an important challenge for PTSD researchers and treatment providers is to understand the dynamic interplay between emotion and cognition. Contemporary cognitive models of PTSD theorize that a preponderance of information processing resources are allocated toward threat detection and interpretation of innocuous stimuli as threatening, narrowing one's attentional focus at the expense of other cognitive operations. Decades of research have shown support for these cognitive models of PTSD using a variety of tasks and methodological approaches. The primary goal of this review is to summarize the latest neurocognitive and neuroimaging research of emotion-cognition interactions in PTSD. To directly assess the influence of emotion on cognition and vice versa, the studies reviewed employed challenge tasks that included both cognitive and emotional components. The findings provide evidence for memory and attention deficits in PTSD that are often associated with changes in functional brain activity. The results are reviewed to provide future directions for research that may direct better and more effective treatments for PTSD.

## Introduction

Stress and anxiety serve the important functions of preparing an individual to meet the demands of everyday life and increasing the chance for survival. It is therefore not surprising that arousing and emotionally salient stimuli readily capture attention and have a powerful influence on how information is processed, encoded, stored, and retrieved. However, extreme levels of stress can have a devastating effect on healthy functioning. Nowhere is this demonstrated more clearly than in psychiatric disorders such as posttraumatic stress disorder (PTSD). PTSD develops after exposure to terrifying and life threatening events and is characterized by intense reliving of the traumatic event through disruptive memories and nightmares, avoidance of reminders of the event, and hypervigilance toward potential threats in the environment. These hallmark symptoms involve alterations to cognitive processes such as memory, attention, planning, and problem solving, underscoring the impact that emotion has on cognitive functioning.

Influential cognitive theories of PTSD emphasize the interaction between emotion and cognition in contributing to the symptoms of PTSD. These theories contend that psychopathology arises when emotional stress alters cognitive networks that process information about perception, meaning, and action responses toward executing goals (Lang, 1977; Foa and Kozak, 1986; Chemtob et al., 1988). In PTSD, networks representing information about fear become highly elaborated and accessible, which has implications for encoding and retrieval of information. For instance, an elaborated fear structure may lower one's capacity to process non-threat related information, leading to attentional bias toward potential threats in the environment (Chemtob et al., 1988). Furthermore, nodes of the fear network representing threat arousal may predispose an individual to interpret even innocuous stimuli as threatening. Intrusive memories result from spreading activation of the threat arousal node to related threat nodes, while nodes representing opposing alternatives become inhibited.

In this review, we summarize the latest research examining the dynamic interplay between emotions and cognitive processes in PTSD. We begin with an overview of the criteria that must be met for a PTSD diagnosis. Next, we separately review studies that examine the effect of emotion on cognitive functions and those that examine the effect of top-down cognitive control processes on emotion, following this useful distinction put forth by Dolcos et al. (2011). Finally, we provide a summary of the reviewed literature and discuss open questions in the field. To directly assess emotion-cognition interactions in PTSD, we focus our review on studies employing a challenge task in which both emotional and neutral stimuli were presented. Such studies may lend themselves to better reproduce conditions in everyday life in which emotions influence task performance. Other recent papers have comprehensively reviewed studies that employed only neutral stimuli (i.e., Aupperle et al., 2012b) or have examined the brain under task-free conditions in PTSD (i.e., Engdahl et al., 2010; Georgopoulos et al., 2010; Daniels et al., 2012).

## Clinical definition of PTSD

As outlined in the current Diagnostic and Statistical Manual (DSM-IV-TR), PTSD develops after exposure to a Criterion A1 event, defined as involving actual or threatened death, serious injury, or threat to one's physical integrity (American Psychiatric Association, 2000). To meet Criterion A1, the individual must have been directly involved in the traumatic event, witnessed the event, or learned about the death or serious injury of a family member or close friend. The individual must have responded to the traumatic event with intense fear, helplessness, or horror (referred to as Criterion A2) although future conceptualizations of PTSD may omit this criterion (Friedman et al., 2011). The symptoms of PTSD can be broadly divided into three symptom clusters, B, C, and D. Symptom cluster B involves persistent and unwanted recollections of the traumatic event, intrusive memories of the event, and dissociative flashbacks. The individual re-experiences the event despite being removed from the traumatic situation and context. These symptoms can be frightening and highly disruptive of activities of daily living. Cluster C involves persistent avoidance of people, places, and activities that serve as reminders of the traumatic event, emotional numbing, difficulty experiencing a full range of emotions, and diminished expectations of one's ability to lead a long, fulfilling life. Finally, symptom cluster D involves symptoms of hyperarousal including difficulty with sleep, irritability and anger, poor concentration, hypervigilance, and exaggerated startle response. The symptoms of PTSD must be present for more than one month and cause significant distress or impairment in social and occupational functioning in order to differentiate the disorder from transient and acute stress reactions. The typical course of PTSD begins with the development of symptoms within 6 months of the onset of the traumatic event, although delays in symptom occurrence can occur. Individuals whose symptoms persist for more than 3 months are diagnosed with chronic PTSD, which is associated with a host of poor health outcomes, including heart disease, obesity, alcohol abuse, and lowered perceptions of general health (Dobie et al., 2004; Hoge et al., 2007; Boscarino, 2008).

The prevalence rate of PTSD is estimated to be 7–8% in the general population (Kessler et al., 1995) although prevalence estimates have varied depending on the type of trauma exposure and demographic characteristics. For instance, prevalence rates are higher among individuals exposed to military combat, ranging between 12–20% (Hoge et al., 2004; Dohrenwend et al., 2006; Tanielian and Jaycox, 2008).

## Emotional effects on cognitive function

### Memory and learning

#### Explicit memory

Decades of emotional memory research in healthy individuals suggests that emotional information tends to be remembered better than neutral information (Christianson, 1992; Kensinger, 2007). However, the extent to which emotion provides a facilitating effect on memory encoding and retrieval in PTSD is unclear. Cognitive models of PTSD predict that patients remember emotional information better due to a bias toward (Chemtob et al., 1988) or difficulty disengaging from (Chemtob et al., 1999) threat-related information, which may lead to greater resources applied to processing and encoding emotional information. A variety of behavioral and neuroimaging memory paradigms have been employed to examine the extent to which patients with PTSD remember emotional information better than neutral information in comparison to healthy or trauma-exposed controls. Consistent with the notion that emotion enhances memory, there is evidence for a memory advantage in patients vs. controls for negative threat information (Vrana et al., 1995; McNally et al., 1998; Golier et al., 2002; Paunovic et al., 2002). In these studies, word lists were presented with either incidental or intentional encoding instructions and participants were subsequently instructed to recall as many words as they could from the lists. Results showed that patients either remembered more emotional words than controls or that memory performance for emotional vs. neutral words improved to a greater extent than controls.

However, memories are often subject to a wide range of distortions and biases that impact accurate recollection (Schacter, 1999). One of the most controversial topics in the field of traumatic stress is that of the accuracy of recovered memories, prompting PTSD researchers to examine how memory for negative and traumatic information fares in false memory paradigms such as the Deese–Roediger–McDermott (DRM) paradigm (Roediger and McDermott, 1995). In the DRM paradigm, participants are presented with a list of words that are semantically related to a critical non-presented word (lure). The critical lure is often falsely remembered as being previously presented on subsequent recall and recognition tests and may reflect gist-based encoding rather than encoding of specific details (Brainerd and Reyna, 2002). In PTSD, two of the three DRM studies employing verbal lists indeed reported greater false alarms to critical lures in patients with PTSD than control participants (Bremner et al., 2000; Brennen et al., 2007). However, a third study employing the DRM paradigm did not report greater false alarms in patients (Zoellner et al., 2000). It is unclear why these studies found differential effects, although it is possible that the false memory effect is more likely to be elicited when trauma-specific material, as opposed to generally negative material, is presented. Studies employing paradigms other than the DRM but including trauma-specific material have reported greater false alarms in PTSD (Hayes et al., 2011) or a bias in making memory decisions about trauma-specific information (Litz et al., 1996).

Negative arousal can alter the type of information that is encoded and retrieved. Neurohormones including norepinephrine and cortisol play a critical role in the fear and stress response by mobilizing the body's response to the stressor via the hypothalamus-pituitary-adrenal axis (HPA) and amygdala, among several other key regions. Norepinephrine has been shown to facilitate emotional memory (for a review see Ferry et al., 1999). However, emotional memory may not be uniformly enhanced during high levels of arousal. For example, individuals exposed to highly arousing negative material show a narrowing of attention (Easterbrook, 1959), referred to as “tunnel memory,” in which the central objects and features of a scene are better remembered than peripheral background (Christianson et al., 1991). A recent study examined the extent to which patients with PTSD showed this memory trade-off effect (i.e., greater memory for negative items vs. backgrounds) in comparison to a trauma-exposed control group and a healthy unexposed group (Mickley Steinmetz et al., 2012). The findings showed that the PTSD and the healthy non-trauma exposed group exhibited a greater memory trade-off effect for emotional items than the trauma-exposed-no-PTSD group. Although further research is required, these results suggest that patients with PTSD do not exhibit greater tunnel memory than healthy control participants.

Distortions in memory have been observed during autobiographical retrieval in PTSD. Autobiographical memories represent personally experienced recollections and knowledge about oneself (Conway and Pleydell-Pearce, 2000) and may be key in understanding the accessibility and completeness of traumatic memories (McNally et al., 1994). Two experimental studies have shown that during the recollection of personal past events, individuals with PTSD tend to recall personal memories with very few details and very little specificity (McNally et al., 1994, 1995). This “overgeneral memory” effect is thought to result from inadequate search of memory during retrieval, perhaps due to rumination, avoidance, and impairment in executive capacity (Williams et al., 2007). However, the difficulty with retrieving detailed personal information does not appear to be specific to traumatic memories but extends to neutral and positive events.

Research on the neural underpinnings emotion and memory suggests that the benefit of emotion on memory occurs in part via interactions between the amygdala and hippocampus. According to the *modulation hypothesis*, emotional events are remembered better than neutral events due to the amygdala's influence on other medial temporal lobe structures including the hippocampus (McGaugh et al., 1996). Support for the modulation hypothesis has been reported in humans using fMRI, showing greater activity in the amygdala and hippocampus for successfully remembered vs. forgotten emotional memories (Dolcos et al., 2004). However, a key question is whether medial temporal lobe structures interact in PTSD as the modulation hypothesis would predict. Whereas the majority of imaging studies have shown increased amygdala activity in PTSD (Pissiota et al., 2002; Shin et al., 2004a, 2005), studies of hippocampal activity have been mixed, showing either an increase (Shin et al., 2004b; Thomaes et al., 2009) or decrease in PTSD (Bremner et al., 2003; Astur et al., 2006). To examine the role of the amygdala and hippocampus in emotional memory formation in PTSD, researchers have employed the subsequent memory paradigm, in which neural activity is measured at encoding for items that are probed for memory success after a delay. Differences in encoding activity for successfully remembered and forgotten material is evaluated to identify brain regions subserving successful memory operations (Paller and Wagner, 2002). Hayes et al. (2011) reported reduced amygdala and hippocampal activity during successful memory encoding of trauma-related material in patients with PTSD. In this study, patients with PTSD produced greater false alarms for trauma-specific negative information, suggesting that the reduced medial temporal lobe activity may underlie memory distortions. However, Brohawn et al. (2010) reported enhanced hippocampal activity in patients with PTSD during encoding of emotional items relative to controls and Dickie et al. (2008) reported greater activity in both the amygdala and hippocampus for remembered vs. forgotten stimuli (a control group was not included in this study and therefore comparisons were made within the PTSD group). Two major differences may explain the discrepant results among studies. In the latter two studies, there were no behavioral differences in memory performance between patients and controls or between emotional and neutral information, and general negative stimuli were used whereas Hayes and colleagues used trauma-specific combat stimuli in recent war veterans. Therefore, although these studies report mixed results, the findings may provide more support for the notion that false memory, and associated decreases in neural signal in the amygdala and hippocampus, is elicited primarily for trauma-specific information in PTSD.

In summary, the research findings of explicit memory performance in PTSD are decidedly complex. The evidence suggests that recall of gist-based negative information may be enhanced in PTSD, whereas information about specific details and contextual information appears to be diminished. This is consistent with the notion that cognitive resources may be preferentially allocated to process threat information at the expense of neutral or non-threat related information. An important consideration is whether memory alterations occur for all types of emotional information or only for trauma-specific information. Although there are mixed findings in this regard, overall there is stronger evidence that false memories are elicited mainly for trauma-specific information. Research regarding the neural correlates of memory in PTSD is still in its infancy, but the abnormalities observed in the amygdala and hippocampus suggests that the symptoms of PTSD are associated with disturbances in memory encoding and retrieval.

#### Fear conditioning

Pavlovian fear conditioning and extinction has been a fruitful model of fear memory in PTSD. Fear conditioning paradigms involve the repeated presentation of a neutral conditioned stimulus (CS) such as an auditory tone or a colored light, followed immediately by an aversive unconditioned stimulus (US) such as a finger shock. Extinction of the fear memory occurs when the CS is subsequently and repeatedly presented in the absence of the US. This experimental paradigm models a crucial aspect of emotion-cognition interactions in PTSD: individuals with PTSD repeatedly show elevated fear responses to trauma reminders, even when those reminders occur in a safe context (i.e., a film portraying combat). Some researchers see a parallel between this clinical phenomenon and a failure of fear extinction or fear extinction recall (e.g., Pitman, 1988).

Early in the PTSD fear conditioning and extinction literature, fear responsivity was quantified as physiological responses such as skin conductance responsivity and heart rate. In line with the enhanced effect of emotion on explicit declarative memory discussed above, physiological studies have demonstrated that, relative to controls, individuals with PTSD show evidence of enhanced fear conditioning (Orr et al., 2000). This may represent a pre-existing vulnerability. Severity of PTSD symptoms and the PTSD-linked personality trait of behavioral inhibition have been correlated with facilitated eyeblink conditioning (Myers et al., 2012). Furthermore, fear extinction studies have demonstrated impaired safety signal learning (Orr et al., 2000; Peri et al., 2000), a possible mechanism for the intractability of fear responses to reminders of a trauma decades past. A more recent study measuring skin conductance responses in identical twins discordant for Vietnam combat exposure provided evidence that deficits in fear extinction recall are an acquired characteristic of PTSD and not a familial risk factor (Milad et al., 2008).

In general, neuroimaging studies of healthy individuals have found activation of amygdala and dorsal anterior cingulate cortex (dACC) during fear conditioning, and activation of ventral medial prefrontal cortex (vmPFC) structures during fear extinction and extinction recall (reviewed in VanElzakker et al., 2012). The first imaging study of fear conditioning in PTSD compared a fear acquisition condition, in which a picture of a blue square was paired with shock, to a control condition, in which participants were shocked randomly without a CS, and found that women with childhood sexual abuse-related PTSD had greater dACC and left amygdala activation than healthy women with no history of abuse (Bremner et al., 2005). During extinction of the blue square-shock association, the PTSD group had less activation in vmPFC structures than the comparison group.

**Figure 1:**
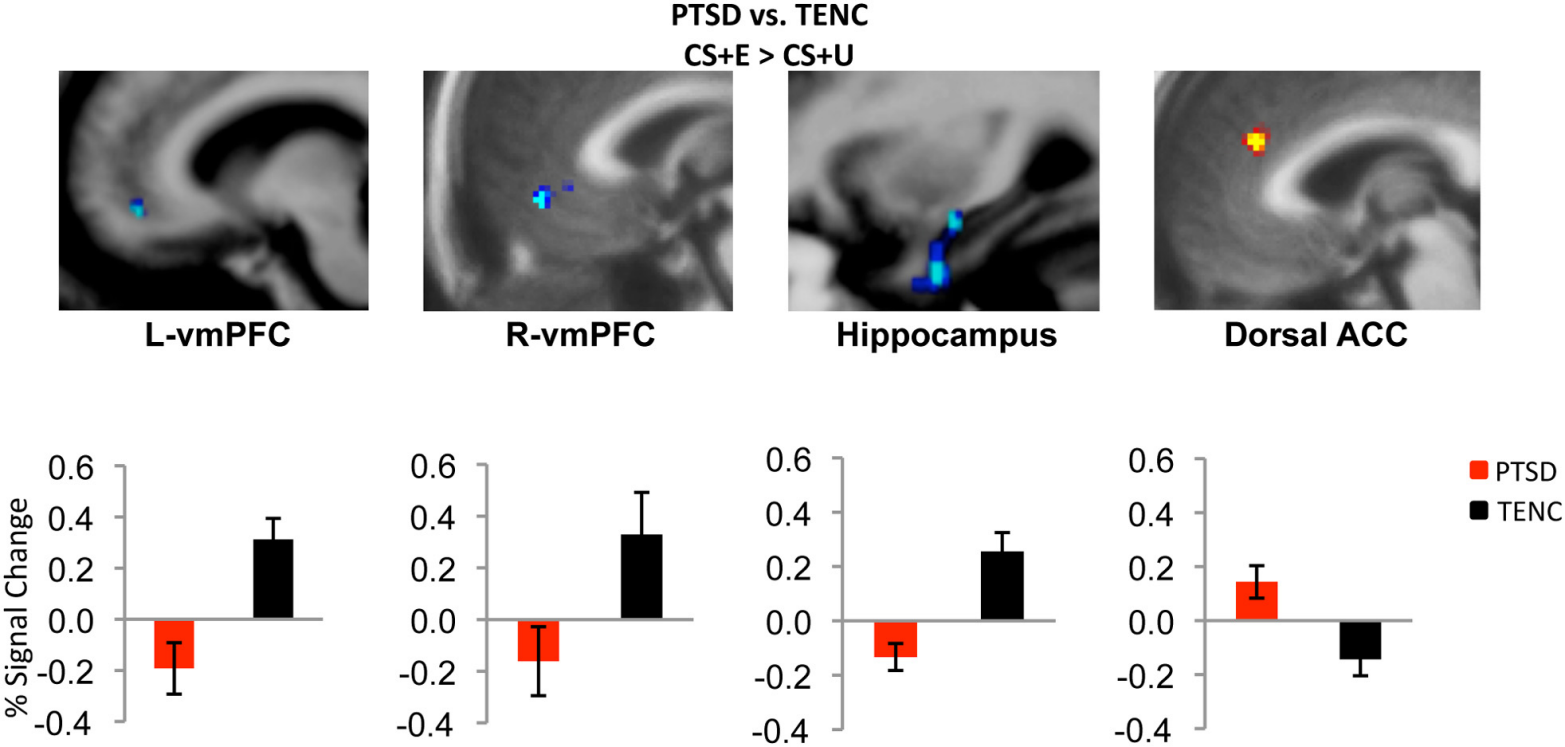
**Neural correlates of extinction recall in PTSD.** During early extinction recall, participants with PTSD showed hypoactivation in bilateral ventromedial prefrontal cortex (vmPFC) and hippocampus and hyperactivation in dorsal anterior cingulate cortex (ACC) relative to the trauma-exposed normal control (TENC) group. CS + E = stimulus that was extinguished; CS + U = stimulus that was not extinguished. Adapted with permission from Figure 3; Milad et al. (2009).

More recently, a series of studies compared fMRI responses between individuals with PTSD and trauma-exposed healthy control participants at each stage of a two-day fear conditioning and extinction paradigm. The authors reported that the PTSD group had increased amygdala responsivity to the UC (shock) relative to trauma-exposed control group (Linnman et al., 2011). During late conditioning and early extinction, after the CS had been associated with the US and still signaled threat, the PTSD group showed increased dACC activation, relative to the control group. Presentation of the CS during late extinction learning, when the CS should no longer have signaled danger, also led to relatively increased amygdala and dACC responses and relatively decreased vmPFC activation in the PTSD group. Furthermore, on the second day of the paradigm, during early extinction recall, the PTSD group showed vmPFC hypoactivation and dACC hyperactivation (Milad et al., 2009; Rougemont-Bücking et al., 2011) (Figure 1).

These results provide a neurobiological basis to the characteristic unrelenting strength of trauma memories in PTSD patients (re-experiencing) and the mechanisms of associative learning between trauma and the environmental cues that later serve as triggers for intrusive memories. The simple fear conditioning model of PTSD does not explain aspects of the disorder such as emotional responses other than fear, neuroendocrine dysfunction, or many of the more complex cognitive deficits discussed elsewhere in this review. However, it has been a valuable model of PTSD in that the functional neurocircuitry abnormalities in this disorder point to enhanced conditioning responses during trauma, as well as deficits in fear extinction and extinction recall after the trauma has passed.

#### Implicit memory

Implicit memory refers to memory for encoded items that are not associated with conscious recollection. Perceptual priming is a type of implicit memory in which prior exposure to a stimulus leads to subsequent facilitated perception of this stimulus. Intrusive memories in PTSD are often triggered by sensory cues that individuals experienced right before or during their traumatic event. Thus, several studies have examined the hypothesis that there is enhanced perceptual priming for trauma-related cues. Early PTSD priming studies used word-stem completion tasks and either failed to find evidence that individuals with PTSD have enhanced priming for trauma-related words (McNally and Amir, 1996) or found only weak evidence (Amir et al., 1996). Using a more sensitive word-stem completion protocol, Michael et al. (2005) found that participants with assault-related PTSD showed preferential priming for assault-related words more than general threat or neutral words, relative to the control group who had experienced assault but did not have PTSD. However, there may be an important reason for the inconsistent findings in word-stem priming studies: words prime conceptual or semantic trauma reminders while the cues that trigger re-experiencing in PTSD tend to be perceptual or sensory. Therefore, perceptual stimuli such as pictures may be more appropriate priming stimuli than conceptual stimuli such as words.

**Figure 2:**
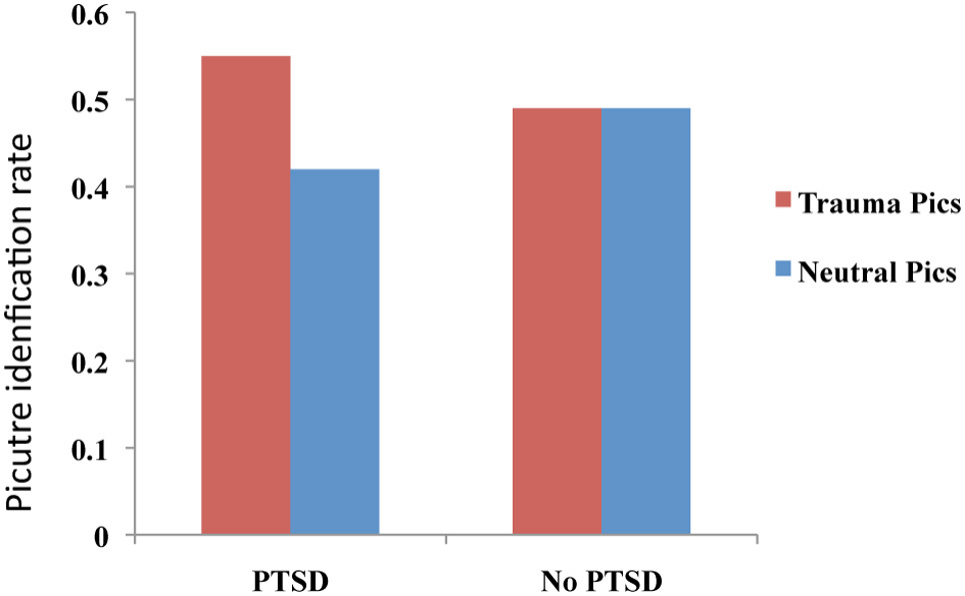
**Performance on an implicit memory task (blurred picture paradigm).** Patients with PTSD identified more trauma-related blurred images than neutral images. No difference between trauma-related and neutral pictures was observed for the No PTSD group. This figure was created from the data supplied in Table 3; Kleim et al. (2012).

A two-part study utilizing a blurred picture paradigm reported that individuals with PTSD and acute stress disorder (ASD) identified more blurred trauma-related pictures than blurred neutral pictures (see Figure 2), and that this processing advantage for trauma-related pictures correlated positively with severity of PTSD symptoms, dissociative symptoms, and re-experiencing symptoms, as well as with self-reports of fear levels and perceptual processing during the actual traumatic experience (Kleim et al., 2012). A follow-up study of the individuals with ASD demonstrated that the initial processing advantage for trauma-related pictures predicted a diagnosis of PTSD 6 months post-trauma.

Blurred picture paradigms have also been used to demonstrate enhanced perceptual priming in healthy, trauma-unexposed individuals for neutral pictures that were associated with violent stories, and an association between enhanced perceptual priming and trait dissociation (Ehlers et al., 2006; Michael and Ehlers, 2007). In addition, “re-experiencing” (operationalized as sensory memories imbued with a sense of immediacy) was associated with greater priming, and that re-experiencing was reduced by asking participants to write about the stories and relate them to their personal lives, which the authors argued was a model of elaboration of traumatic memories during clinical therapy. Future studies may replicate these findings in participants with PTSD.

In a recent fMRI study of trauma-unrelated emotional priming, Mazza et al. (2012) administered a subliminal affective priming task to 10 individuals with earthquake-related PTSD and to 10 healthy controls. In this task, the subliminal (150 ms) presentation of emotional faces was immediately followed by the supraliminal (1850 ms) presentation of neutral pictures (Chinese ideographs), which were later rated as pleasant or unpleasant. Individuals with PTSD were more likely than controls to rate the ideographs that followed negative facial expressions as negative, and less likely than controls to rate the ideographs that followed positive facial expressions as pleasant, suggesting a propensity in PTSD for priming of threat-related cues. Furthermore, during the contrast between negative facial expression primes and the baseline fixation dot, individuals with PTSD had significantly greater BOLD responses in left amygdala and right insular cortex, relative to controls.

In summary, some priming studies demonstrate enhanced perceptual priming for threat-associated cues in both individuals with PTSD and in healthy populations. The enhanced priming for threat cues may be associated with hyperactivity of amygdala and insular cortex in PTSD. Future studies should more systematically investigate differences in conceptual vs. perceptual priming. The propensity for enhanced perceptual priming of these cues, combined with the facilitated conditioning and attenuated fear extinction recall discussed previously, may be a powerful combination in both the etiology and maintenance of PTSD.

### Attention and working memory

#### Attention bias

In individuals with PTSD, trauma-related memories intrude into consciousness and are difficult to ignore. In addition, reminders of traumatic events can capture attention and evoke distress and anxiety. Some researchers have hypothesized that in PTSD, attention is involuntarily biased toward stimuli that are threatening, leading to a disruption of ongoing cognitive activities. Researchers have attempted to study attention biases in the laboratory using several different paradigms: the emotional Stroop (also known as the “modified Stroop”), the dot probe paradigm, and the emotional oddball paradigm.

In the emotional Stroop, researchers ask participants to view (on a computer screen or on printed cards) words of varying emotional salience and to name the color of the words while ignoring their meaning. The researchers record the time it takes for participants to name the colors of different types of words (e.g., trauma-related words, neutral words, positive words). Delays in color naming (i.e., Stroop interference) occur when the meaning of a particular category of words is closely related to the participants' psychopathology and thus attracts attention despite efforts to the contrary.

Several studies in the literature have reported increased response times for trauma-related words relative to neutral words (and/or other types of words) in individuals with PTSD compared to trauma-exposed comparison participants without PTSD (e.g., McNally et al., 1990; Foa et al., 1991; Cassiday et al., 1992; Thrasher et al., 1994; Bryant and Harvey, 1995; Kaspi et al., 1995; Vrana et al., 1995; Beck et al., 2001; El Khoury-Malhame et al., 2011a). Furthermore, measures of trauma-related Stroop interference have been shown to positively correlate with PTSD symptom severity (McNally et al., 1990; Cassiday et al., 1992; Paunovic et al., 2002; Fleurkens et al., 2011). Stroop interference in PTSD appears to be specific to trauma-related material, although some studies have reported interference to other types of emotional stimuli (Litz et al., 1996; Paunovic et al., 2002). Increased interference for trauma-related words in PTSD may not occur outside of conscious awareness, as this effect has not been consistently demonstrated with masked stimulus presentations (McNally et al., 1996; Paunovic et al., 2002) but see also (Harvey et al., 1996). Importantly, however, some studies have failed to replicate the finding of greater interference for trauma-related words in PTSD (e.g., Freeman and Beck, 2000; Devineni et al., 2004; Wittekind et al., 2010) see also (Kimble et al., 2009).

Two functional neuroimaging studies have attempted to examine the brain circuits that may mediate emotional Stroop interference in PTSD (Shin et al., 2001; Bremner et al., 2004). Both studies reported that the rostral anterior cingulate cortex (rACC) was less activated during trauma-related vs. control Stroop conditions in individuals with PTSD compared to trauma-exposed individuals without PTSD. One of the studies also found greater activation in the dACC in PTSD during trauma-related vs. generally negative Stroop conditions (Shin et al., 2001). Rostral ACC activation may be required to effectively ignore the trauma-related information in the service of completing the color-naming (or word-counting) task at hand. When the rACC is not functioning normally, increased activation of the dACC may be required in order to facilitate task performance. Interpretation of these imaging findings within the framework of earlier behavioral findings in the literature is somewhat limited by the fact that response times in the neuroimaging studies were either not measured (Bremner et al., 2004) or did not show significant group differences (Shin et al., 2001) probably due to small sample sizes.

Although a useful tool for investigating the nature of intrusive cognitions in PTSD, the emotional Stroop is limited in that it cannot be used to determine whether individuals with PTSD have increased attentional engagement to trauma-related stimuli or delayed disengagement from them. The dot probe task (sometimes called the attentional deployment task, the visual probe task, or the probe detection task) represents an improvement over the Stroop task in that it can measure the direction of attentional bias (e.g., toward or away from trauma-related stimuli) as opposed to merely assessing the existence of interference, and can also use pictorial stimuli, reducing the need for semantic processing (MacLeod et al., 1986). In the dot probe task, two stimuli (e.g., one trauma-related and one neutral) are briefly shown on either side of a screen. The participant responds when a target probe then appears in the location previously occupied by one of the stimuli. Attentional bias toward trauma-related stimuli would result in faster reaction time during those trials in which the probe replaces the trauma-related stimulus.

Dot probe studies in PTSD have reported mixed findings. Some studies have found bias toward trauma or threat-related stimuli in PTSD (Bryant and Harvey, 1997; Dalgleish et al., 2001, 2003; Fani et al., 2012), while others reported an association between PTSD and a bias away from trauma or threat (Pine et al., 2005; Fani et al., 2011). Still others have failed to find significant attentional bias differences between PTSD and two control groups, consisting of healthy individuals and a group of recent trauma survivors that included both individuals with and without ASD (Elsesser et al., 2004, 2005).

A recent fMRI study of the dot probe task presented angry (threat-related) and happy and neutral (threat-unrelated) faces to female survivors of multiple traumas who either did or did not have PTSD (Fani et al., 2012). Within the PTSD group but not within the control group, bias toward threatening faces correlated positively with activation in the dACC and insula, as well as the parietal lobe, caudate and the medial frontal, precentral and parahippocampal gyri. However, there were no response time differences between the two groups, indicating no consistent bias toward or away from threat.

Time since trauma may be an important factor in these inconsistencies. There is evidence that, under immediate acute stress conditions, individuals under threat have a bias away from threat-related stimuli, which predicts later PTSD symptoms (Wald et al., 2011). One series of studies tested this explicitly, under unique circumstances. During the Israeli military operation against Gaza (Operation Cast Lead) Israeli civilians near the border experienced a predictable increase in danger from retaliatory rocket attacks. The immediacy of danger increased as a function of proximity to border areas, allowing for quantification of threat. Using a dot probe task adapted for Hebrew, researchers found that individuals under greatest imminent threat had an attentional bias away from threat-related words (e.g., DEAD) compared to neutral words (e.g., DATA) (Bar-Haim et al., 2010). Individuals who were more than 40 km from the border and not within rocket range showed attention bias toward threat-related words. PTSD and depression symptoms also increased as a function of threat, and state anxiety was highest among individuals who lived within 10 km of the border and thus had 15 s or less to seek shelter when they heard warning sirens. One year after the conflict, attentional bias away from threat during the acute stressor predicted PTSD symptoms (Wald et al., 2011). The process by which attentional bias away from threat during an acute stressor putatively transforms to attentional bias toward threat in PTSD may be related to the “rebound effect” discussed below in the section concerning thought suppression.

Attentional bias toward threat in PTSD could reflect either difficulty disengaging from threat-related stimuli or facilitated engagement of such stimuli. A study of healthy individuals that related attentional mechanisms to subclinical PTSD symptoms provided indirect evidence that attentional bias toward threat in PTSD reflects difficulty disengaging as opposed to facilitated engagement (Bardeen and Orcutt, 2011). Interestingly, difficulty disengaging from threatening stimuli has been associated with the 5-HTTLPR serotonin transporter gene polymorphism (Beevers et al., 2009). Children's 5-HTTLPR short allele significantly moderated the relationship between maternal criticism and the children's attentional bias for angry faces, but not happy or sad faces, in a dot probe task (Gibb et al., 2011). This same polymorphism may predict poor response to cognitive-behavioral therapy in PTSD (Bryant et al., 2010). Furthermore, an fMRI study of a related attentional task called the detection of target (DOT) paradigm demonstrated that amygdala activation in PTSD patients, but not in healthy controls, correlated with attentional bias toward threatening faces and words (El Khoury-Malhame et al., 2011b). Future studies can better elucidate the relationships among time since trauma, serotonin function in the amygdala, and attentional processes in PTSD.

The emotional oddball paradigm has proved to be useful in demonstrating attention bias in PTSD. In this task, infrequent target stimuli are interspersed with frequent standard stimuli and infrequent distractor emotional stimuli. The task requires participants to inhibit their prepotent response to frequent standard and distractor stimuli in order to identify the target stimuli accurately. Patients with PTSD are impaired in identifying neutral targets, which may be a consequence of attention bias to distracting, potentially threat-related information (Pannu Hayes et al., 2009). Furthermore, event-related potential (ERP) studies have shown that during processing of threat stimuli, an enhanced P3 amplitude response is observed in patients with PTSD, which is thought to reflect heightened attention toward those stimuli (Attias et al., 1996; Stanford et al., 2001). More recently, fMRI studies using the emotional oddball paradigm demonstrated that PTSD symptomatology was associated with greater activity in the dorsolateral prefrontal cortex and vmPFC for threat stimuli (Pannu Hayes et al., 2009), accompanied by a reduction in dorsolateral prefrontal cortex activity for target, non-threat stimuli (Morey et al., 2008; Pannu Hayes et al., 2009). These studies provide a neural marker for threat bias in PTSD that is characterized by heightened activity in putative attention and emotion circuitry for potentially threatening information and dysfunction in attention circuitry during goal-relevant target identification.

Anticipation of an impending negative stimulus may influence attention allocation and subsequent cognitive performance. Researchers have examined whether women exposed to intimate partner violence show alterations in attention performance and neural circuitry while anticipating a negative visual stimulus (Simmons et al., 2008; Aupperle et al., 2012a). One particular study with a large sample size (41 women with PTSD and 34 healthy controls) examined the neural correlates of negative and positive anticipation embedded within a continuous performance task (Aupperle et al., 2012a). Results indicated that patients with PTSD showed greater activity in the insula and less activity in the dorsolateral prefrontal cortex than controls during anticipation of negative events. Furthermore, greater activity in the dorsolateral and ventrolateral prefrontal cortex was associated with better performance on an attention switching task (i.e., the Color–Word Interference Inhibition/Switching subtest of the Delis–Kaplan Executive Function System) and a digit symbol test. These intriguing results may suggest that engaging the lateral prefrontal cortex in the face of anticipatory threat supports cognitive performance, possibly through an inhibitory mechanism.

In summary, a majority of studies have found evidence for attentional bias effects in PTSD. Although the findings are mixed, there appears to be growing evidence that the attentional bias reflects difficulty disengaging from, rather than facilitated detection of, negative stimuli. Collectively, the brain regions consistently active during tasks of negative attention in PTSD include the dACC, amygdala, insula, with mixed findings of the vmPFC. The aforementioned regions have previously been associated with emotional reactivity, perhaps underlying privileged processing of negative images in PTSD.

#### Working memory

Working memory is often defined as the maintenance and manipulation of information in a temporary memory store (Baddeley, 1992). Importantly, working memory has a limited capacity, suggesting that individuals can track and work with a small amount of information at a given time. An implication of this limited capacity store is that interference from distracting stimuli can reduce an individual's ability to maintain goal-relevant information. The interference of distracting stimuli, such as intrusive thoughts and trauma memories seems to be a particular difficulty in PTSD and may underlie the hallmark symptom of difficulty with concentration. Working memory deficits in patients with PTSD have been demonstrated using both verbal and visual stimuli. Schweizer and Dalgleish (2011) reported poorer working memory performance in patients vs. trauma-exposed controls on a verbal sentence task, in which participants were instructed to remember words presented following trauma-related or neutral sentences. Consistent with the idea that trauma-related material is particularly disruptive to working memory performance, memory was worse for words presented after trauma vs. neutral sentences. Working memory difficulty was observed in both participants with a current diagnosis of PTSD and individuals with a lifetime history of PTSD.

Neuroimaging studies investigating the impact of emotional distraction on working memory have suggested that hyperactivity in an emotional processing network (including regions such as the amygdala, ventrolateral prefrontal cortex, and medial prefrontal cortex) and hypoactivity in a dorsal executive function processing network (including regions such as the dorsolateral prefrontal cortex and parietal cortex) underlies impaired maintenance of information in working memory as a result of emotional distraction (Dolcos and McCarthy, 2006). This model was supported in an fMRI studying examining working memory in PTSD. Morey et al. (2009) showed that patients with PTSD had poorer memory performance when both neutral and trauma-specific distracters were presented during the working memory delay in comparison to a trauma-exposed control group. Furthermore, this fMRI study showed disrupted activity in the dorsal executive function network during the working memory delay in PTSD that could explain the diminished performance. An interesting outcome of this study is that performance was disrupted for both trauma-specific and neutral distracters, perhaps providing evidence for generalized hypervigilance.

### Decision-making and reward processing

Individuals make decisions in part based on motivational influences, weighing the rewards and costs that may result from each option. On one end of the spectrum, seeking immediate positive rewards is associated with the psychopathology of addiction disorders (Bechara et al., 2002) while on the other end of the spectrum, lack of reward seeking is associated with depressive disorders (Pizzagalli et al., 2008). In PTSD, numbing symptoms including loss of pleasure in activities and loss of the ability to experience positive emotions may suggest altered processing of positive rewards. Consistent with this notion, patients with PTSD are less satisfied with rewards than controls (Hopper et al., 2008) and expend less effort to obtain positive rewards (Elman etal., 2005). Thus, it follows that patients with PTSD may have altered decision-making capacity if the drive to achieve positive rewards is reduced.

Neuroimaging studies in healthy individuals have supported the notion of a putative reward circuit that includes the ventral striatum, ventral pallidum, orbital frontal cortex, and anterior cingulate. Two studies have examined the neural correlates of decision-making and reward in PTSD, both providing evidence for reduced capacity for positive reward in PTSD. Sailer et al. (2008) showed that the nucleus accumbens (part of the ventral striatum) was less active in patients with PTSD than controls during processing of positive gains. Behaviorally, patients with PTSD were slower in learning how to maximize their gains in a monetary gain/loss paradigm. Although speculative, it is possible that reduced reward processing in PTSD may have negatively influenced patients' motivation in learning the task. Similarly, PTSD patients showed reduced activity in the striatum during gains vs. losses of a monetary task in another study (Elman et al., 2009). Interestingly, striatal activity for gains vs. losses was negatively correlated with CAPS items “loss of interest in significant activities” and “feelings of detachment/estrangement.”

## Cognitive control of emotion and treatment effects

When distressing events occur during the course of the lifespan, individuals often engage in various strategies to manage and cope with negative emotions. In doing so, they can change their emotional experience of the stimulus, and also change how the emotional stimulus affects their cognitive performance. Individuals can exercise control of emotion for the desired effect; they can engage in cognitive reappraisal to improve affect, or conversely, amplify negative affect (Dillon et al., 2007). Furthermore, cognitive control strategies can be used to improve memory performance (Hayes et al., 2010) or suppress unwanted memories (Depue et al., 2007). Such deliberate modulation of emotion serves to manage distractions in the face of otherwise debilitating affect and helps individuals to remain focused on goal-directed behaviors. In treating psychopathology, cognitive-behavioral interventions often instruct patients to exercise greater control of emotion through thought challenging exercises and promoting alternative ways of thinking about a negative situation (Resick and Schnicke, 1993). Preliminary evidence suggests that these types of interventions not only reduce symptoms, but also improve cognitive performance (Sutherland and Bryant, 2007).

In PTSD, experimental work investigating the cognitive control of emotion has examined whether patients can purposely forget negative information. Some researchers have suggested that patients with PTSD have an enhanced ability to forget information, which may explain amnesia for important details of their traumatic event. This idea, mainly evolving out of the child sexual abuse literature, suggests that repeat trauma survivors with PTSD cope during their trauma by dissociating from their surroundings and disengaging attention from the event, sometimes leading to amnesia for large stretches of time (Terr, 1991). This avoidant coping style may manifest in adulthood by increased ability to forget new information presented in an experimental setting (McNally et al., 1998). Alternatively, others have suggested that intrusive memories arise from the failure of inhibitory processes to curb distracting and aversive memories (Zwissler et al., 2011).

One frequently used paradigm to examine this issue is the directed forgetting task, which examines deliberate attempts to control memory performance. In this task, participants are instructed to either remember or forget words presented in a list. Subsequently, participants attempt to recall and/or recognize all the words that were presented in the list regardless of whether they were to-be-remembered or to-be-forgotten. The “standard directed forgetting effect” refers to the behavioral outcome showing greater remembering for items presented during the remember condition than the forget condition. The task is often adapted for study of PTSD by including general emotional or trauma-specific words. Two studies that examined directed forgetting of trauma-relevant information in PTSD failed to show between group differences among PTSD and control participants (McNally et al., 1998; Zoellner et al., 2003). However, these studies and others have demonstrated differences between groups for non-trauma emotional and/or neutral stimuli. Specifically, patients fail to show the standard directed forgetting effect either due to difficulty forgetting to-be-forgotten items (Zoellner et al., 2003; Cottencin et al., 2006) or decreased performance in the remember condition (McNally et al., 1998; Zwissler et al., 2011). Taken together, the directed forgetting literature provides little evidence that patients with PTSD adopt an avoidant coping style that results in enhanced forgetting of negative information. Rather, a general inhibitory control mechanism may be impaired evidenced by poorer performance for non-threat related items.

**Figure 3:**
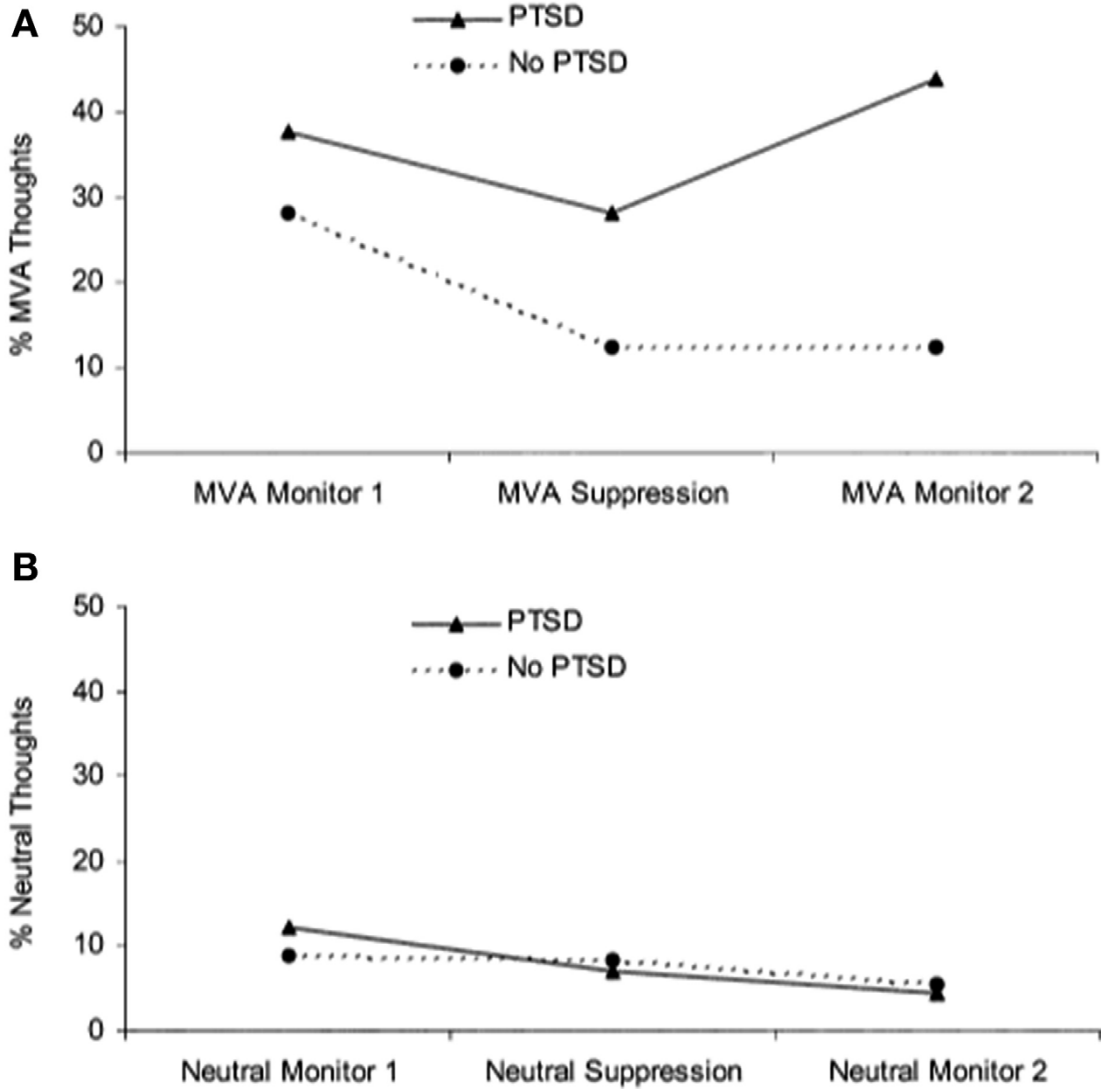
**Performance on a thought suppression task. (A)** Patients with PTSD reported more trauma-related (i.e., motor vehicle accident, MVA) thoughts after engaging in thought suppression, showing a “rebound” effect. (**B**) Participants did not show a rebound effect for neutral thoughts. Reproduced with permission from Shipherd and Beck (2005).

A separate literature has emerged examining the effects of actively suppressing one particular thought. Unlike directed forgetting paradigms, thought suppression is less concerned with recall and recognition memory performance but rather the frequency with which thoughts arise following instructions to suppress them. Wegner and colleagues demonstrated that when participants were instructed to initially suppress a thought, they went on to think about it to a greater extent than if they were initially allowed to let the thought enter consciousness, referred to as a “rebound effect” (Wegner et al., 1987). Two studies have shown that patients with PTSD have more trauma-related thoughts after a thought suppression period than trauma-exposed controls (Shipherd and Beck, 1999, 2005) (see Figure 3). In another study that instructed participants to suppress neutral information, combat veterans with PTSD had greater combat-trauma related intrusions during attempts to suppress thoughts about a “white bear” than combat veterans without PTSD (Aikins et al., 2009). These studies suggest that attempts at thought suppression might in fact be associated with greater frequency of trauma-related cognitions in PTSD.

Other forms of cognitive control include emotion regulation strategies, in which participants are instructed to change their natural response to a stimulus. Gross and colleagues have shown that individuals can engage in thought-change strategies, such as cognitive reappraisal, to deliberately reduce negative affect (Gross, 1998). Most studies examining emotion regulation in PTSD have used self-report measures to examine the frequency with which these different strategies are used and whether they are associated with greater or reduced frequency of trauma symptoms. However, there appears to be only one paper to date that has directly manipulated emotion regulation strategies in PTSD (New et al., 2009). This fMRI study compared sexual assault victims with and without PTSD on an emotion regulation task. Participants were shown negative photos and instructed to down-regulate their emotional response to the picture (diminish condition), enhance their negative response, or maintain their current response to the picture. Behavioral results showed that the healthy control group was able to diminish negative affect to a greater extent than the PTSD group whereas no group difference was observed in the enhance condition. Imaging results showed that the control group recruited greater prefrontal cortex activity across superior and middle frontal gyri for both the diminish and enhance conditions, which may suggest that controls engage in cognitive control to a greater extent than patients with PTSD.

Psychosocial treatment interventions may benefit patients by teaching them the cognitive control skills necessary to manage their symptoms, thereby reducing the detrimental effect of strong negative emotion on cognitive performance. Alternatively, drug therapies may directly affect neural circuitry and consequently blunt the effect of emotion on cognitive function. A few studies have examined the extent to which therapy improves cognitive function in PTSD. Although the evidence is limited, there is a small body of data that supports the effectiveness of therapy on normalizing cognitive function in certain domains in patients. Sutherland and Bryant (2007) reported improved autobiographical memory specificity in PTSD after treatment. Two studies have reported improved emotional Stroop performance in patients relative to controls after psychosocial intervention (El Khoury-Malhame et al., 2011a; Thomaes et al., 2012). Finally, Putman et al. (2007) reported a reduction in color naming response times to masked fearful vs. neutral facial expressions after a 40 mg dose of hydrocortisone (vs. placebo) in highly anxious men. Although this study did not include individuals with PTSD, its findings seem to call for examining the effect of glucocorticoids on emotional Stroop interference in PTSD, especially given that the administration of glucocorticoids has been associated with symptomatic improvement (e.g., Surís et al., 2010) and a reduction of fear responses in this disorder (e.g., Jovanovic et al., 2011; Miller et al., 2011), but see also (Grossman et al., 2006). Other studies, however, have not found effects of treatment on emotional Stroop measures in PTSD (Devineni et al., 2004; Taylor et al., 2006). Clearly, additional research is necessary to examine what types of treatments may confer benefits in cognitive function to individuals with PTSD.

## Summary and future directions

The literature summarized here provides strong support for the privileged processing of emotionally charged information in PTSD. A key question is whether emotional information facilitates or interferes with cognitive processing. In other words, does PTSD confer advantages in cognitive performance given that emotional stimuli are often processed with greater efficiency than neutral stimuli? Over the span of different study paradigms, there appears to be a trade-off in cognitive performance as cognitive models of PTSD predict; although fear learning, perceptual priming, and recall memory for negative items are sometimes enhanced in PTSD, this advantage comes at the expense of processing other types of information. For example, task-irrelevant emotional information slows processing of goal-directed activity and interferes with memory and learning of neutral information. Furthermore, extinction learning and learning of safety cues is often impaired, memory for specific, detailed information is often poor, and patients with PTSD may be more prone to falsely remembering novel information. Deficits in cognitive control and emotion regulation may be exacerbated by the impact of emotion on cognitive function.

**Figure 4:**
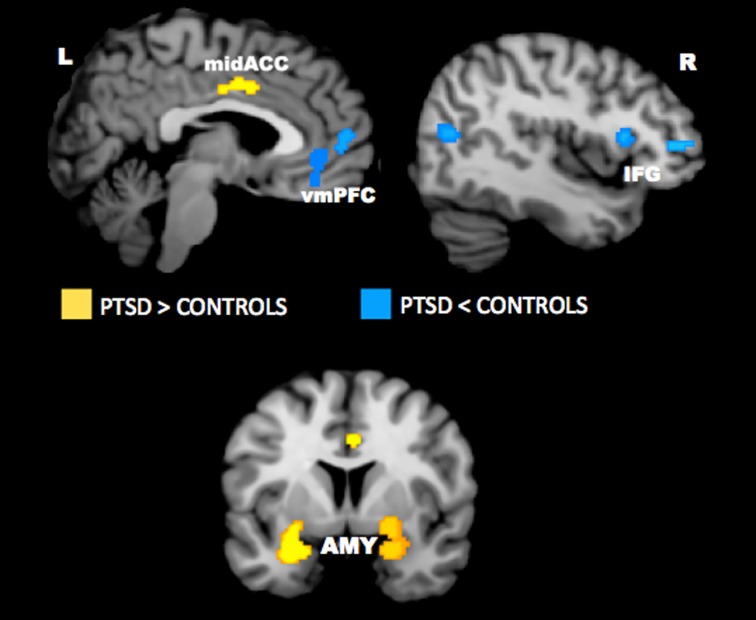
**Meta-analysis of functional neuroimaging studies in PTSD.** Across various task designs, the amygdala and mid-ACC are hyperactive in PTSD whereas the lateral and medial prefrontal cortex are hypoactive for negative emotional stimuli vs. neutral and positive stimuli. Areas of hyperactivation in PTSD (PTSD > Control) are shown in yellow and areas of hypoactivation in PTSD (Control > PTSD) are shown in blue. AMY = amygdala, IFG = inferior frontal gyrus, L = left, mid-ACC = mid anterior cingulate cortex, R = right, vmPFC = ventromedial prefrontal cortex. Reproduced from Hayes et al. (2012).

Neuroimaging studies have uncovered several key brain regions that may underlie the emotional bias effects observed in PTSD. Across studies, activity appears to be altered in the anterior cingulate cortex, vmPFC, amygdala, hippocampus, insula, and lateral prefrontal cortex. Findings from quantitative meta-analyses of the neural correlates of PTSD have confirmed the importance of these regions in PTSD (Etkin and Wager, 2007; Hayes et al., 2012; Simmons and Matthews, 2012). A recent meta-analysis of imaging studies in PTSD showed that the amygdala and mid-ACC is hyperactive, whereas lateral and medial prefrontal cortex is hypoactive in PTSD for negative emotional stimuli vs. neutral and positive stimuli (Figure 4). A neurocircuitry model of PTSD posits that dysfunction of the vmPFC prefrontal cortex results in failure to inhibit an overactive amygdala, leading to an exaggerated fear response and impaired fear extinction learning (Rauch et al., 2006). Hippocampal dysfunction may be related to impairment in processing contextual information (Rauch et al., 2006; Hayes et al., 2011). The dACC, anterior insula, and amygdala, among other regions, comprise a putative “salience network” that processes information of personal relevance and is hyperresponsive in individuals with anxiety (Seeley et al., 2007). Researchers have posited that, in PTSD, hyperresponsivity of salience network regions and hyporesponsivity in putative regions important for cognitive control and working memory underlie greater distribution of processing resources in favor of potentially threatening stimuli even when neutral information is goal-relevant (Morey et al., 2009; Pannu Hayes et al., 2009; Hayes et al., 2012).

As reviewed in this paper, our understanding of emotion-cognition interactions in PTSD has progressed tremendously over the last two decades and neuroimaging research has identified pathways involved in the effect of emotion on cognition. However, a number of key questions need to be further examined to gain a better understanding of how emotion affects cognitive function in PTSD. An important consideration is the extent to which individual differences and moderating factors impact the development of PTSD. There is some evidence that deficits in configural cue processing and lower IQ precede the development of PTSD (Gilbertson et al., 2007; Vasterling et al., 2002). Further research is necessary to determine whether impairment in other cognitive processes precede trauma exposure and, conversely, the extent to which cognitive control and emotion regulation capacity prior to trauma exposure can offer resilience.

Currently, little is known regarding the neurobiology of cognitive control in PTSD. Surprisingly few studies have examined the neural correlates of deliberate attempts to control emotions. As reviewed above, there is presently only one neuroimaging study investigating emotion regulation in PTSD (New et al., 2009). This area needs to be developed to further examine whether impairment in the cognitive control of emotions is a PTSD symptom-maintaining factor and whether neural abnormalities during top-down regulation of emotion represents a useful biomarker for the diagnosis of PTSD. Moreover, further examination of the interplay between medial temporal lobe structures and the prefrontal cortex is necessary to better understand control processes during emotional memory encoding and retrieval. In healthy individuals, prefrontal cortex activity is associated with deep semantic encoding that supports improved recall of emotional memories (Ritchey et al., 2011), as well as suppression of aversive memories that reduces recall (Depue et al., 2007). However, less is known regarding the extent to which patients with PTSD can engage critical prefrontal cortex regions to influence the memorability of emotional stimuli.

Another emerging area of research is using drug therapies to manipulate emotion-cognition interactions. Norepinephrine has been shown to enhance emotional memory whereas adrenergic receptor blockers such as propranolol compromise the enhancing effect that emotional arousal has on memory (Cahill et al., 1994). Cerebrospinal norepinephrine levels are elevated in chronic PTSD (Geracioti et al., 2001) and research regarding the effectiveness of adrenergic blockers in preventing PTSD is underway. Although preliminary results have not yielded strong evidence to recommend the use of propronolol for the prevention of PTSD (Pitman et al., 2002; Vaiva et al., 2003; Hoge et al., 2012), further research is necessary to determine the precise time window in which such drug therapies may be useful (Cain et al., 2012). Furthermore, additional research is required to examine whether these drugs impact specific types of memory (e.g., explicit vs. implicit memory, autobiographical vs. memory for general negative events, central vs. peripheral details).

Finally, a very important area that is understudied is the commonalities or specificity of cognitive alterations in PTSD vs. other comorbidities such as depression, traumatic brain injury, and attention deficit and hyperactivity disorder. In many cases, cognitive abnormalities are observed across different mood and anxiety disorders. For example, overgeneral autobiographical memory is also a characteristic of individuals diagnosed with depression. Although many studies of PTSD focus on the fear and anxiety based symptoms such as hypervigilance, it is possible that many of these cognitive deficits are related to the dysphoria and maladaptive appraisals that are often observed in PTSD. In fact, in recognition of these common symptoms, the proposed changes to the current diagnosis of PTSD for DSM-V may add a fourth symptom cluster of “negative alterations in cognitions and mood” (Friedman et al., 2011). Furthermore, a movement toward dimensional classification of symptoms that are shared among disorders is gaining momentum (Ofrat and Krueger, 2012). It is therefore important to understand the similarities and differences in emotion-cognition interactions between PTSD and other comorbidities to better determine the origin of and potential treatments for PTSD.

### Conflict of interest statement

The authors declare that the research was conducted in the absence of any commercial or financial relationships that could be construed as a potential conflict of interest.
